# The Status of the Plastic Surgery Workforce for Women and Recommendations on How to Be Successful

**DOI:** 10.1093/asjof/ojad027.008

**Published:** 2023-04-14

**Authors:** Paige Benyamein, Sandhya Kalavacherla, Lucy Sheahan, Emily Ewing, Katerina Gallus, Anureet Bajaj, Amanda Gosman

**Affiliations:** University of California San Diego, San Diego, CA; University of California San Diego, San Diego, CA; University of California San Diego, San Diego, CA; Rady Children's Hospital, San Diego, CA; Restore SD Plastic Surgery, San Diego, CA; Bajaj Plastic Surgery, Oklahoma City, OK; University of California San Diego, San Diego, CA

## Abstract

**Goals/Purpose:**

The status of women in medicine has been rapidly evolving. Recent data shows that women now make up the majority of current medical students in the United States. Women now constitute 40.5% of integrated plastic surgery residents and make up 20% of the current plastic surgery workforce. The evolving face of the plastic surgery workforce warrants a deeper look at where we stand today. There has been literature published in recent years looking at women plastic surgeons in academia but little attention has been paid to the greater female plastic surgery workforce to include those in other practice settings.

The purpose of this study is to look at current practices for female plastic surgeons, paths taken to get there, and advice for young female surgeons and surgical trainees. We aim to characterize and project future trends of the status of women in plastic surgery. We further outline recommendations on how to be successful as a female entering the field.

**Methods/Technique:**

A survey was created using Survey Monkey with a series of 37 questions. These questions focused on demographics, what practices currently look like, paths taken to get there, individual desires for practice changes, and advice for female plastic surgeons early in their career. Email addresses were obtained through a web-based search using both the American Society of Plastic Surgeons and American Society of Aesthetic Plastic Surgeons listed board-certified female plastic surgeon members in the United States. A total of 748 surveys were emailed and survey responses were collected.

**Results/Complications:**

There was a total response rate of 33%. Most respondents trained in an integrated pathway program (54%) and did not complete a fellowship (37.7%). The majority practice in a large city (44.6%), work 40-60 hours per week (63.4%) and perform 20-30 cases per month (34.05%). Most respondents work in a solo practice (32.05%). For those that previously worked in a different practice setting, the most common prior settings were small group practice (36.89%) and academia (32.04%). Fifty six percent of women did not have a female mentor. The majority of respondents are very satisfied in their current career (54.63%). The factors that ranked highest for helping women achieve success were persistence (81.14%), patient care (76.75%), patient rapport/communication (77.19%) and grit (74.89%). The highest ranked barrier to success included discrimination as a female (17.18%), lack of mentorship (16.81%), and personal/family demands (16.74%). Recommendations for women pursuing a career in private practice included starting to learn to run a business in residency/fellowship and get additional business education, finding a mentor, and to start growing your social media platform during training.

**Conclusion:**

The plastic surgery workforce for women continues to evolve. Most practicing female plastic surgeons recommend for trainees to start learning how to run a business during their training and acquire additional business education. In addition, they stress the importance of mentorship throughout this process. As more women continue to enter the field of plastic surgery, mentorship and support for childcare to help with family demands will be paramount to their success. Residency programs should consider incorporating more business training into their curriculum given the majority of trainees will go on to work in a private practice setting.

